# *Schools as Hubs of Health*: A Comprehensive Supplemental Nutrition Assistance Program—Education Model for Promoting Wellness in Low-Income Communities

**DOI:** 10.3390/children11050525

**Published:** 2024-04-27

**Authors:** Shannon A. Klisch, Katherine E. Soule

**Affiliations:** 1 University of California Cooperative Extension in San Luis Obispo & Santa Barbara Counties, University of California, Agriculture and Natural Resources, 2156 Sierra Way, Suite C, San Luis Obispo, CA 93401, USA; 2 Department of Nutrition, University of California, Davis, 3135 Meyer Hall, One Shields Avenue, Davis, CA 95616, USA

**Keywords:** nutrition education, social determinants of health, SNAP-Ed, policy, systems, and environmental changes, social ecological model

## Abstract

Research indicates that health interventions are most effective when they address multiple social determinants of health to support positive behavior. *Schools as Hubs of Health*, a comprehensive model of nutrition and physical activity education, was developed to support wellness within school communities defined as low-income by the national Supplemental Nutrition Assistance Program Education (SNAP-Ed). Components of the model include the following: classroom education; garden education; youth engagement; staff training; parent and community engagement; and policies, systems, and environments. Findings over the last decade indicate positive outcomes in nutrition and physical activity behaviors, youth leadership and engagement, and systems and environmental changes that support health and wellness.

## 1. Introduction

There are indicators that overall dietary quality among U.S. children is improving; however, 49% continue to have poor diet quality [1]. Further, the average adolescent (aged 9–13) does not meet recommended intake levels for fruits, vegetables, whole grains, and dairy and more than 75% exceed recommended dietary limits for added sugars, saturated fats, and sodium [2]. Poor diet is a major contributor to diabetes, heart disease, stroke, and cancer, accounting for substantial chronic disease morbidity and premature mortality [3]. Conversely, the consumption of fruits and vegetables has been shown to decrease the risk of cancer, stroke, depression, and cardiovascular disease [4]. While these outcomes are typically measured in adulthood, dietary habits developed in youth shape lifelong food preferences [5] and, therefore, can impact health outcomes later in life. In addition, increasing physical activity is associated with reductions in all-cause mortality, type 2 diabetes, and many other health conditions that impact quality of life [6].

One of the largest federal programs intended to improve dietary quality and physical activity among low-income Americans is the Supplemental Nutrition Assistance Program Education (SNAP-Ed). SNAP-Ed is administered and regulated by the Food and Nutrition Service within the US Department of Agriculture (USDA), with the goal to “improve the likelihood that persons eligible for SNAP will make healthy food choices within a limited budget and choose physically active lifestyles consistent with the current Dietary Guidelines for Americans [7]”. Since the Healthy, Hunger-Free Kids Act of 2010 [8], SNAP-Ed has expanded from evidence-based individual and group education to incorporate support for broader public health approaches through policy, system, and environmental changes (PSEs). The uptake of PSE approaches by states has been incremental and a need for more research and translational science on PSE implementation and evaluation has been identified in the literature [9].

Schools have been identified as an effective setting for preventing obesity and promoting healthy behaviors [10,11]. Changes in school food policy due to the 2010 Healthy, Hunger-Free Kids Act coincided with large and equitable improvements in the dietary quality of food consumed from schools among U.S. children [12]. However, a review of school nutrition standards showed that improvement in access to healthier options did not guarantee an improved selection and consumption of these options [13] and that further interventions were needed in conjunction with policy changes to ensure students benefit. At the same time, hands-on, skills-based, and interactive nutrition education interventions have been associated with increases in students’ knowledge, attitudes, and perceptions; however, behavioral and secondary outcomes (e.g., body mass index) have been mixed [14] possibly due to the fact that changes to the environment and to access have not been addressed.

This is in line with the U.S. Department of Health and Human Services [15] seeking to address the significant contribution of environmental influences on individual and community health disparities through social determinants of health. By focusing on social determinants of health, including equitable access to a solid academic foundation, nutritious food, and environmental conditions that support wellbeing, researchers and scholars found that youths’ access to these supportive factors directly impacts socio-emotional and physical wellbeing [16,17,18].

With research indicating that health interventions are most effective when they address multiple levels of influence, such as individual, family, environmental, and/or policy [19] nutrition educators, public health practitioners, school administrators, and decision makers have utilized the socio-ecological model and their understanding of social determinants of health with increasing frequency to understand contributors to health behaviors [20] and to implement effective programming aimed at changing environments and influencing behaviors to improve health [21].

Increasingly, there has been an emphasis on multi-component or comprehensive school-based interventions to improve health behaviors or health outcomes [22], evidencing that comprehensive school health interventions, which include policy or other school environment modifications and incorporate multiple components, can improve dietary behaviors and body composition [23,24]. Several recent review articles have highlighted qualities of school-based nutrition and physical activity programs that improve health behaviors or outcomes. These include coordinated approaches (school wellness councils, committees, or teams to address physical activity and/or nutrition) [25]; professional development or technical assistance [24,26]; family, parental, or community engagement [26,27,28]; physical activity policies; improvements to physical education and recess interventions [29]; and experiential nutrition education to improve skills (cooking, gardening, taste-testing, etc.) combined with other strategies [27].

This article describes the efforts over 10 years across five school environments to incorporate multi-component nutrition and physical activity interventions to improve dietary behavior and student wellness among students attending low-income schools in two California counties. This paper adds to the literature regarding applied research in SNAP-Ed program delivery as experienced by researchers overseeing the program design and implementation at the University of California Cooperative Extension. This adds to the literature on translation science in PSE implementation in the SNAP-Ed context.

In 2021, the Cooperative Extension’s National Framework for Health Equity and Well-being was released, which coordinates efforts around the social determinants of health to address inequities through Cooperative Extension research and education, including SNAP-Ed [30].

### 1.1. Background

Since 2014, the University of California’s Cooperative Extension Youth, Families, & Communities Program in San Luis Obispo & Santa Barbara Counties (YFC) has implemented a comprehensive model of nutrition and physical activity education called *Schools as Hubs of Health.* The *Schools as Hubs of Health* model supports wellness, health, and thriving within school communities defined as low-income by SNAP-Ed. To positively influence health for this population, the YFC Program led the development, implementation, and evaluation of research-based educational programming that provides a wide range of health-focused interventions, targeting multiple levels of influence in five school communities through SNAP-Ed program delivery. Building on the Whole School, Whole Community, Whole Child model from the Centers for Disease Control and Prevention [31], *Schools as Hubs of Health* involves comprehensive nutrition education and obesity prevention school wellness services.

The primary goal of *Schools as Hubs of Health* is to support improved nutrition and physical activity behaviors and overall wellness among students and families through (1) increasing knowledge and awareness of healthy behaviors; (2) engaging youth leaders in becoming advocates for health and wellness in their schools and communities; and (3) facilitating changes in school policies, systems, and/or physical environments. Over the past decade, the model has undergone continuous, iterative development to identify community needs and opportunities, and to refine programming and delivery elements in partnership with five school communities in two school districts in the Santa Barbara and San Luis Obispo Counties of California. In the 2023/24 academic year, these schools serve a combined 3952 students annually with a predominantly Hispanic or Latino (94%) youth population, where the majority of students are English Learners (68%) and qualify for free or reduced-price school meals (89%).

### 1.2. Theoretical and Conceptual Frameworks

In a recent review of the literature on the effectiveness of school-based interventions to promote healthy lifestyle behaviors, researchers noted that effective interventions included at least two of the following components in their design: teachers’ training, parental involvement, and a theoretical model. On the contrary, unsuccessful school-based interventions were not based on a well-defined theoretical framework. Additionally, the inclusion of a theoretical model in the intervention programs led to the improvement of other parameters, mostly in dietary behavior [32].

The *Schools as Hubs of Health* model for comprehensive nutrition education services was developed based on the Social Ecological Framework for Nutrition and Physical Activity Decisions [33] to positively address social determinants of health in specific school communities and incorporates several theoretical models. The social ecological framework requires program planners to consider broader societal determinants in program design and to look beyond individual behaviors to understand environmental and contextual barriers and facilitators to health. Building off these frameworks, *Schools as Hubs of Health* was designed to consider the interactions between individuals and families with physical, social, cultural, and policy environments at school and in their communities. Additionally, the model incorporates multiple theoretical frameworks in the educational approach and delivery, which are described in alphabetical order.

#### 1.2.1. Asset-Based Community Development

Building on existing assets (including local knowledge, culture, resources, skills, and interests) of the participants and community, this delivery model recognizes the community and participants as experts and leaders in the movement towards increasing community health [34]. In this delivery model, the approach is closely connected to community participatory action research and supports improvements and health within the participating school communities in partnership with students, school personnel, families, and community members. Over the last decade, the *Schools as Hubs of Health* model emphasized sustained engagement with partnering school districts, administrators, teachers, families, other community-based organizations, and students to identify and address health and wellbeing, building on the students’ and communities’ existing strengths, interests, and assets.

#### 1.2.2. Health Equity

Seeking to ensure that all individuals have the opportunity, resources, and support to experience their highest level of health, this model incorporates health equity principles into program development and delivery [35]. From site selection to environmental supports, policy improvements, and evaluation, the *Schools as Hubs of Health* program considers what schools, communities, and youth need to thrive.

#### 1.2.3. Positive Youth Development

Utilizing the positive psychological approach, positive youth development (PYD) supports positive outcomes as youth age through opportunities for skill development, cultivating caring relationships, access to positive environments, increased social networks, and improved self-esteem and efficacy [36]. With an emphasis on increasing protective factors in youths’ lives, the *Schools as Hubs of Health* program intentionally provides a multi-faceted PYD approach to improve participants’ wellbeing during program participation and into adulthood.

#### 1.2.4. Social Cognitive Theory

Recognizing the interplay “between people (personal factors), their behavior, and their environments” [37], *Schools as Hubs of Health* incorporates Social Cognitive Theory (SCT)’s personal cognitive, socio-environmental, and behavioral constructs into program design and delivery. *Self-efficacy* and *knowledge* build youth confidence to try new foods through exposure, social modeling, and practice in stress-free conditions along with instruction around healthy behavior (personal cognitive constructs). *Observational learning* through peer-to-peer education is delivered by youth leaders, addressing *barriers and opportunities* through community-driven changes in the school environment and support for classroom teachers to include research-based nutrition and physical activity curricula in their lessons (socio-environmental constructs). *Skill building* around cooking and growing food is taught through hands-on lessons throughout the school year, and *intentions* include goal setting in the classroom (behavioral constructs). While SCT is an important individual behavior change theory, “SCT focuses primarily on individual behavior change [and] environmental influences are often overlooked or not adequately considered in intervention design”, and the depth of SCT’s impact on individual behavior change is “enhanced by the breadth of the socioecological model” [37] through the *Schools as Hubs of Health* model.

#### 1.2.5. Trauma Informed Pedagogy

Understanding that exposure to trauma can significantly impact individuals’ ability to learn [38] and that vulnerable populations are more likely to have experienced traumatic events [39], this pedagogical approach emphasizes communication techniques, classroom management strategies, and relational approaches that center student learning to support students who have experienced trauma, traumatic stress, and/or traumatic grief. *Schools as Hubs of Health* staff are trained in trauma-informed pedagogical approaches, including in communication techniques, classroom management, and relational approaches.

## 2. Materials and Methods

**Figure 1:**
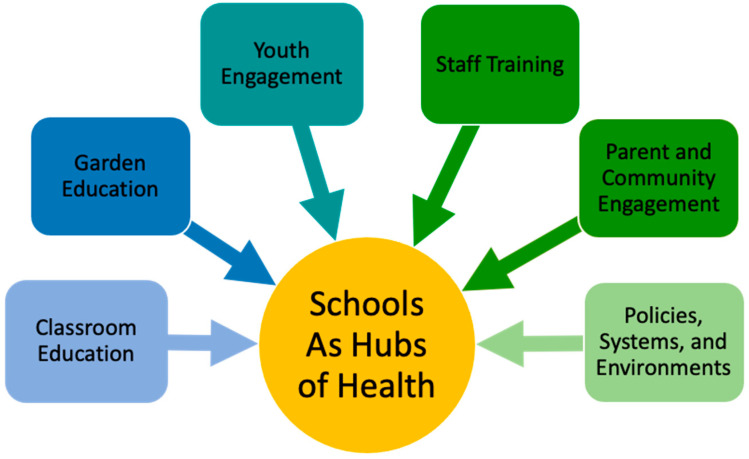
*Schools as Hubs of Health* comprehensive model of nutrition and physical activity education.

*Schools as Hubs of Health* incorporates interventions that reach priority populations at multiple levels of the social ecological model in school settings. In alignment with USDA SNAP-Ed Guidance from 2013–2024, the model includes behavior-focused education and support for behavior change through multicomponent interventions. The approaches within the *Schools as Hubs of Health* model incorporate evidence-based group direct education (DE), multicomponent communication to reinforce direct education or indirect education (IE), and/or policy, systems, or environmental change interventions (PSE) to support healthy behaviors. Specifically, components of the *Schools as Hubs of Health* delivery model include classroom education (DE); garden education (DE, PSE); youth engagement (DE, IE, PSE); staff training (DE, PSE); parent and community engagement (DE, IE); and policies, systems, and environments (IE, PSE) (see Figure 1). Each of these components are described in the following sections. All individual research participants were provided written informed consent with information describing the research being conducted, that their participation was voluntary, that they could choose to discontinue their participation in the data collection at any time without any negative consequences, and who to contact for questions or concerns. With younger participants, informed consent was read to them by a trained data collector. Participants that continued with the data collection were considered consenting based on a protocol reviewed by the UC Davis Institutional Review Board (see specific numbers for each collection tool below).

### 2.1. Classroom Education

Classroom education (DE) was delivered by elementary (transitional-kindergarten through 6th grade) classroom teachers and SNAP-Ed program staff. This education included evidence-based grade-level nutrition and physical activity curricula, cooking demonstrations and skill-building, and food tastings to support TK-6 curriculum objectives. Staff training is described below in Section 2.4.

Evaluation of the classroom education component included the following: tracking participant numbers and activity duration; collecting participant feedback to assess learning and make improvements to future component implementation via qualitative feedback surveys; and three evaluation tools discussed here. The Eating and Physical Activity Tool for Students (EATS) utilizes pre- and post-evaluations collected from fourth-grade youth who met the inclusion criteria. Youth were included if they consented to participate, were enrolled in a classroom that received a minimum of six hours of DE as reported by teachers and program staff, and were present in class when the evaluation surveys were collected. The EATS tool was developed and validated by the UC Nutrition Policy Institute. The Taste Test Tool (TTT) was developed and validated by CalFresh Healthy Living, UC, to evaluate youth’s willingness to try and request new foods in tastings that occur within classroom education. The Teacher Observation Tool (TOT) asks about teacher perceptions of nutrition or health-related behavior changes in youth or themselves compared to the beginning of the school year. The TOT was developed for CalFresh Healthy Living, UC, and is administered with teachers enrolled in SNAP-Ed programming. The UC Davis Institutional Review Board reviewed and approved the protocol for collecting these data (IRB ID#235428-23).

### 2.2. Garden Education

The garden education component (DE, PSE) included (1) enhancing school gardens, (2) conducting garden-based nutrition education, (3) development and delivery of educator curriculum kits for garden use, and (4) supporting garden-to-cafeteria connections. The YFC program supported school garden development and/or enhancement at all *Schools as Hubs of Health* sites and partnered with community-based organizations to train local teachers and volunteers about garden-enhanced nutrition education.

Evaluation of the school garden components included the following: tracking the number of garden sites supported annually, collecting participant feedback to assess learning and make improvements to future component implementation via qualitative feedback surveys; tracking the pounds of produce harvested from school gardens for use in the cafeteria or for families to eat at home; and implementing environmental scan tools to identify needed garden-related PSE changes that were a priority for school communities.

### 2.3. Youth Engagement

The youth engagement component (DE, IE, PSE) focused on developing student leaders through the afterschool 4-H Student Nutrition Advisory Council (4-H S.N.A.C. Club) [40,41]. The goals of the 4-H S.N.A.C. Club are to (1) develop student leaders in nutrition and physical activity in order to create healthy schools and communities, (2) establish positive youth–adult partnerships in order to improve youth outcomes related to health and academics, and (3) increase access to 4-H among underserved Hispanic or Latino communities. 4-H S.N.A.C. Club student leaders were trained in leadership and presentation skills, nutrition and MyPlate, food safety, and physical activity. 4-H S.N.A.C. Club meetings occurred weekly after school and hosted two annual educational events during academic breaks.

Evaluation of the youth engagement component included the following: tracking participant numbers and activity duration; collecting participant feedback to assess learning and make improvements to future component implementation via qualitative feedback surveys; and three evaluation tools discussed here. Leadership, positive youth development, and health behavior outcomes were evaluated using the Youth Healthy Living pre/post-survey from 2015/16 to 2017/18 and the Youth Leader Retrospective (also known as the Teen Teacher Retrospective) Survey from 2018/19 and on. Both tools are based on the 4-H Common Measures for Healthy Living, Citizenship, and Youth Development [42], which were evaluated for reliability and validity by the California 4-H Youth Development program. In 2018/19 and 2019/20, youth also completed a self-report retrospective program evaluation. The 2015/16 protocol was reviewed by the UC Davis Institutional Review Board (IRB ID#717416-1). The remaining Youth Healthy Living and Youth Leader Retrospective data collection methods were reviewed and approved by the UC Davis Institutional Review Board (IRB ID#235428-23).

### 2.4. Staff Training

The staff training component (DE, PSE) includes training for classroom teachers, P.E. Specialists, lunchroom, and afterschool staff. Staff training included (1) modeling curriculum delivery, MyPlate nutrition messages, and food safety for teachers; (2) CATCH P.E. training to improve youth participation in moderate to vigorous physical activity through inclusive P.E. lessons; (3) garden-based nutrition education; and (4) training and technical assistance for Smarter Lunchroom Movement interventions.

Evaluation of the staff training component included the following: tracking participant numbers and activity duration; collecting participant feedback to assess learning and make improvements to future component implementation via qualitative feedback surveys; outcomes of staff training were measured using a self-report survey where participants report knowledge gained and estimate when they plan to use the information provided, as well as the retrospective Teacher Observation Tool (TOT) described above; and implementing environmental scan tools to identify needed PSE changes that were a priority for school communities. The UC Davis Institutional Review Board reviewed and approved the protocol for collecting these data (IRB ID#235428-23).

### 2.5. Parent and Community Engagement

The parent and community engagement (DE, IE) component includes schoolwide family health nights, food bank distribution educational activities, parent education nights and class series, parent letters to promote family goal setting and alignment with the nutrition curriculum, career days with local professionals, mass media engagement, and opportunities for youth leaders to present their vision for community health improvements to decision makers and elected officials. YFC program staff partnered with schools to provide virtual family cooking nights and class series in Spanish and English and to maintain social media accounts in both English and Spanish for parents to obtain information about nutrition, food security, physical activity, and cooking. Professionals from the community—including dentists, engineers, health educators, college professors, and firefighters—taught students about college and career pathways during career fairs organized by YFC program staff. YFC program staff created educational videos for families and community members. The program also engaged with local policy and decision makers, including youth presentations at school board meetings; city council meetings, principal meetings and for the superintendent; conducted garden tours; and involved speaking at youth educational events.

Evaluation of the parent and community engagement components included the following: tracking participant numbers; collecting participant feedback to assess learning and make improvements to future component implementation via qualitative feedback surveys; and findings from parent and community engagement evaluations were previously reported [22]. The UC Davis Institutional Review Board reviewed and approved the protocol for collecting these data (IRB #213961-13).

### 2.6. Policies, Systems, and Environments

The policies, systems, and environments (PSE) component is integrated throughout all other components of the model. This component builds upon the leadership of youth developed through the 4-H S.N.A.C. Club, the access to school garden produce supported through the school garden component, and partnerships with classroom teachers and P.E. Specialists, school administrators, and cafeteria staff.

Evaluation of the PSE components included implementing environmental scan tools including the Shaping Healthy Choices School Health Check (2016/17–2018/19), the Smarter Lunchrooms Movement Scorecard (2014/15–2016/17), and the Site-Level Assessment Questionnaire (2019/20, 2022/23) to identify needed PSE changes that were a priority for school communities, as well as tracking observed changes in policies, systems, or environments over a fiscal year period through a state-level tracking system. Program staff who were tracking PSE changes received detailed training and guidance through state SNAP-Ed staff about how to report PSE changes.

## 3. Results

### 3.1. Classroom Education

More than 20,000 youth participated in Schools As Hubs of Health since program development (2014/15–2022/23). These youth participated in over 6200 hours of direct education through the program. Findings from classroom evaluations are shared in the tables below. (Please note: due to the COVID-19 pandemic, evaluation outcomes were not completed in 2019/20 or 2020/21.)

#### 3.1.1. Taste Test Tool Findings

Outcomes from the TTT indicated that most students showed a willingness to try new foods that were presented in the classroom and a willingness to ask for the food at home (Table 1).

#### 3.1.2. Teacher Observation Tool (TOT)

Outcomes from the TOT indicated that at the end of the school year, more students could identify healthy food choices, were willing to try new foods, and would choose fruits and vegetables to eat during classroom parties than compared to the beginning of the year (Table 2).

#### 3.1.3. Eating and Physical Activity Tool for Students (EATS)

EATS data collection beginning in 2022 indicated the percentage of students who reported an increase in healthy food consumption, a decrease in sugary beverage consumption, and an increase in physical activity (Table 3).

#### 3.1.4. Quotes and Reflections from Qualitative Feedback Surveys

Reflecting on the classroom education, teachers shared:

- “Most of the kids said that they had only previously tasted white flour tortillas, not wheat tortillas. They were surprised that it tasted so good. The majority indicated that they would inform their parents as to the health benefits of choosing wheat tortillas over regular flour tortillas”.
- “The students loved trying the beets in a smoothie. Mixing it with fruit in a smoothie challenged them to try new things”.
- “Today in class we tasted fruits and vegetables some of them we like and some we didn’t”.
- “We made a veggie stir fry it tasted delicious”.
- “I notice that more students are eating the healthy choice. [YFC] is making a difference on our campus”.

Student shared:

- “Thank you for letting us try the garbanzo beans on their own, I never knew I would like them so much by themselves with nothing on them. I am going to look for them on the salad bar next time!”
- “I loved (the Brussels Sprouts) so much I’m going to go ask the (Lunch Staff) if we can have these on our lunch line so I can eat them again!”

### 3.2. Garden Education

The *Schools As Hubs of Health* program has supported the building or reinvigoration of between 3 and 14 school and community gardens each year, providing opportunities for youth to spend time outdoors, learn about nutrition, and grow food. Since tracking began in 2020, over 2400 pounds of produce have been harvested from school gardens and donated to the school meal program, local families, and/or local food pantries. Additionally, the *Schools as Hubs of Health* program supported food service in two school districts to develop and implement written food safety protocols for school-grown produce in the cafeteria, which increased access to garden-to-cafeteria produce for 27,000 low-income students. Many garden education impacts are identified in the PSE results section below, as well as captured in the EATS and TOT data reported in classroom education and staff training above.

Quotes from student leaders involved in teaching in and maintaining their school garden about the best part of the program from qualitative feedback survey:

- “Learning new stuff for cooking and garden. It has helped my garden”. (2023)
- “Getting to teach younger kids about Ag the way I wish I would’ve been taught”. (2023)
- “The best part was being able to learn new things about plants while being able to teach others about new things. Also being able to eat what we grew”. (2022)

### 3.3. Youth Engagement

From academic years 2014/15 to 2022/23, 778 youth leaders in 4th through 6th grades participated in 4-H S.N.A.C. Club training, reaching over 11,000 youth through peer-to-peer teaching in the classroom, cafeteria, school garden, and family and community education. Positive outcomes reported include the following:

- Increased positive health behaviors, leadership, teaching, cooking, gardening, and self-confidence among participants.
- Notable outcomes include increased nutrition knowledge, behavior changes, and improved skills in presentation, leadership, and cooking.
- Post-program surveys indicated significant improvements in healthy behaviors, such as choosing water over sugary drinks and consuming smaller servings of high-fat foods.
- Youth development measures revealed enhanced decision-making abilities, community involvement, and leadership skills.
- Statistically significant positive youth development outcomes were documented, including gains in public speaking, program planning, and teaching.
- Participants reported knowledge gains and anticipated behavior changes related to leadership and peer teaching.
- Notable impacts from the 2021/2022 academic year include enhanced leadership skills, improved health behaviors, and positive qualitative feedback highlighting the program’s benefits.

Overall, the findings underscore the program’s effectiveness in fostering positive youth development and promoting healthy behaviors among participants. Additionally, student leaders shared via qualitative feedback surveys:

- “I like the nutrition club. It helps me to eat healthy food. It also helps me to do exercise every day. I also like how we do a lot of fun things.”
- “I like to jump rope. They let us use knives. And they let us eat. And they let us plant. And they let us cook.”
- “We got to exercise and meet new people. We also go to tell other people about [4-H S.N.A.C. Club] and how it’s important and useful. It’s really fun being in [4-H S.N.A.C. Club] because we get to do presentations, exercise, cook healthy, and make healthy things.”
- “I enjoy seeing other kids’ expressions trying new foods. I also like to talk to my family about it.”

### 3.4. Staff Training

Each academic year from 2014/15 to 2022/23, between 94 and 322 educators participated in Schools As Hubs of Health and provided over 5800 h of nutrition education and physical activity services across all academic years. During these academic school years, 377 staff completed self-reports reporting that 98.6% learned new information as a result of their participation in staff training, and 97.6% anticipated positive behavior change as a result of their participation. Findings from the Teacher Observation Tool (TOT) (see Table 4), which were completed annually at the end of the school year, are shared in the table below. (Please note: due to the COVID-19 pandemic, evaluation outcomes were not completed in 2019/20–2021/22.)

Additionally, staff training participants shared the following via qualitative feedback surveys:

- “The experience of engaging and empowering youth to make healthier choices and to also teach their peers to make healthier choices has been very encouraging and rewarding. I have witnessed many individual and group successes from the youth that we are working with within a short time period. Many of the youth we have worked with have gained an abundance of confidence in expressing their knowledge of nutrition, health, and physical activity and building their skill set for public speaking and project development. They are becoming more comfortable taking the initiative on creating and developing the projects that are most important to them”.
- “The things [students] learned are really sticking with them. They are reading nutrition labels and identifying healthier choices”.
- “Very informative! I learned things I didn’t know, surprised at what I did know. This was fun”.
- “I would also like to somehow teach students to take only what they can eat. To not take so much that they have to toss food”.
- “Enjoyed learning how to make food look better and how to make the lunchroom environment better. I think it’s great that we are working on bettering our lunch site”.
- “Good ideas to bring back to our work force. Always good to have a fresh look at things. Wish I would have taken your workshop sooner”.
- “Excellent ideas to get kids to eat more by appealing visuals”.
- “Idea of having input from students i.e., food names. I will give it a try!”
- “I feel like small changes can be made for big results”.

### 3.5. Parent and Community Engagement

More than 3200 parents and community members participated in direct nutrition education and 67,500 people received indirect education through *Schools As Hubs of Health* since program development in 2014/15. After attending the parent and family education sessions, adult survey respondents reported positive changes in their approach to feeding their children. Some results from parent education have been reported previously, including positive parent feeding behaviors from pre to post. “The behaviors that changed the most from pre to post included not offering a treat for eating food (66.67% reported positive change), children eating snacks at the same time every day (33.33% reported positive change) and serving foods to kids again after they reject it the first few times (91.67% reported positive change)” [43]. Below are participation counts from parent and community engagement virtual educational programming:

More than 19,500 people were reached through the programs’ English and Spanish Facebook and Instagram pages in the last two years, which provides information and links to healthy recipes, food safety information, physical activity tips, and information about Schools as Hubs programming.

More than 42,000 views were gained on YouTube across 33 nutrition and physical activity educational videos developed by program staff for family and community members, which include lessons on how to grow food at home, learning about healthy recipes, and getting enough physical activity.

Additionally, parents shared the following via qualitative feedback surveys:

- “I like how it tastes and I would make this for my family”.
- “I’ve changed many things at home. My children found it fun when I shared how to properly clean hands, fruit, and countertops. Everything was fun. Some of my favorite techniques that I learned: how to prevent spread of bacteria, how to organize the fridge, use different cutting boards. I enjoyed learning how to take care of my home and family. Thank you!”
- “I have learned to pay more attention to what I’m eating and what I’m feeding my son. There is a variety of foods that I like that are healthy for me so now I substitute those foods with the unhealthy foods I like”.
- “A mi familia nos ha cambiado mucho la manera de como comer saludable y mas economico. Menos gastamos en comprar cosas malas. Mis hijos les gusta lo que les han ensenado en sus clases que se queda con este programa, y por eso me gusta porque mis hijos ya no les gusta comer cosas que no son saludables. Gracias por este programa, esta muy bien ayudando a nuestros hijos”. [English translation: My family has changed a lot about the way we eat healthier and more economical. We spend less money on buying bad things. My children that are with your program, like what they have been learning in their classes. I like the program because my children no longer like to eat things that are not healthy. Thanks for this program, it’s very good and helps our children].
- “Aprendemos comer más saludable y estar más sano con la familia”. [English translation: We learned how to eat healthier and to be a healthier family].
- “Aprendo tomar mucha agua y consumar la menor azucar possible”. [English translation: I learned to drink more water and to consume as little sugar as possible].
- “Aprendí que comer frutas y verduras es saludable en la vida, para los niños es muy importante”. [English translation: I learned that eating fruits and vegetables is healthy for your life, and is very important for the kids].

### 3.6. Policies, Systems, and Environments (PSE)

Environmental scan tools were utilized to identify PSE changes that were a priority for school communities. Examples of PSE changes that were supported include the following: supporting students and/or parents to learn in, eat from, and/or work in their school garden; supporting efforts to incorporate and promote school garden produce in the cafeteria; increasing access to physical activity opportunities including active recess and classroom brain breaks; increasing access to safe and appealing drinking water through promotion and/or installation of hydration stations, and Smarter Lunchroom Movement evidence-based interventions in school cafeterias. The table below shows the number of PSE changes supported at community or school sites, including school-district-wide improvements (Table 5). PSE reach was determined and reported by staff working at individual sites based on standardized guidelines developed by the CalFresh Healthy Living, UC state agency, in collaboration with the California Department of Social Services.

## 4. Discussion

Improving youth health and wellbeing requires interventions at multiple levels of influence, ranging from individual to family to environmental to policy. *Schools As Hubs of Health* utilizes a comprehensive approach to addressing the environmental, systemic, and social constraints that impact youth, family, and community health. This program draws on community strengths, youth leadership, and strong partnerships to support improvements in the health of people living in the communities surrounding these schools. In general, it is difficult to compare this comprehensive school-based SNAP-Ed intervention with non-school SNAP-Ed interventions due to lack of consistent data collection and reporting. However, a recent systematic review of coordinated, multicomponent physical activity and/or nutrition interventions in schools found 21 unique studies describing 24 interventions [25]. These interventions included changes to the nutrition environment, physical education, parent engagement, community engagement, and nutrition or physical activity curricula components. The authors found that results from multicomponent interventions varied across studies, with some positive findings related to the school environment or student behaviors or outcomes. This is consistent with our findings that the Schools as Hubs of Health model can promote improved dietary behavior and improvements in the school environment. Further, this study incorporates the additional aspects of positive youth engagement and garden education to report on outcomes related to youth leadership and self-confidence. While this paper describes applied research in a live setting and is not as scientifically rigorous as the randomized control trials and quasi-experimental studies covered in Chung et al. 2023 [25], we believe it has merit related to informing program planners and SNAP-Ed implementers about the potential impact of this type of multi-component programming. Further, as most of this programming was funded through SNAP-Ed, one of the largest and most sustainable sources of nutrition education funding in the nation, we believe this program warrants further study and a more rigorous study design in the future.

### 4.1. Limitations

While the Schools As Hubs of Health program has demonstrated considerable success, several limitations and challenges merit acknowledgment. These include the following:

- Challenges in evaluating comprehensive, longitudinal programming, particularly in capturing the cumulative impacts of multifaceted interventions.
- Difficulty in assessing changes in behavior over time due to the dynamic nature of youth development and the lack of consistent data collection methods.
- Limited comparability between school-based SNAP-Ed interventions and non-school interventions, hindering comprehensive evaluation and analysis.

### 4.2. Future Research

Beyond program implementation and delivery, there are significant opportunities for future research to enhance program evaluation methodologies and advance understanding in key areas. To effectively demonstrate the profound impact of comprehensive and longitudinal programs on community health and wellbeing, future research should focus on refining evaluation methodologies, which is essential for articulating the broader societal benefits of such programs.

#### 4.2.1. Evaluation of Comprehensive, Longitudinal Programming

- Current evaluation tools available to SNAP-Ed implementers may not adequately capture the overall impacts of such programming. Future research should focus on developing and refining evaluation methodologies to better quantify the cumulative impacts of comprehensive interventions.
- Longitudinal studies that track youth from early grades (T-K) through sixth grade can provide valuable insights into the long-term effects of school-based interventions. However, existing pre/post-evaluation tools may not be sufficient in capturing developmental changes in behavior over time.
- Comparative research between school-based SNAP-Ed interventions and non-school interventions is needed to assess their respective reach and impact. Standardized data collection and reporting protocols should be established to facilitate meaningful comparisons across different intervention settings.

#### 4.2.2. Evaluation of Policy, System, and Environmental (PSE) Efforts

- Environmental scan tools play a crucial role in identifying priorities and initiating conversations with stakeholders. However, their effectiveness in measuring changes over time at site or institutional levels may be limited.
- Future research should explore innovative approaches to evaluating PSE efforts, with a focus on developing more precise and comprehensive measurement tools.

#### 4.2.3. Community Engagement in Evaluation Processes

- While current evaluation tools may be meaningful to funders, there is a need to incorporate additional measures that are relevant and meaningful to program participants, partners, and communities.
- Greater community engagement in the evaluation planning process is essential to ensure that evaluation indicators align with the needs and priorities of stakeholders.
- Efforts to address evaluation fatigue and minimize participant burden are crucial for maintaining engagement and participation in longitudinal evaluation studies.
- Evaluation planning should prioritize the inclusion of measures that resonate with youth, families, and school administrators, thus providing a more holistic understanding of program impacts.

### 4.3. Implications for Practice

In light of the program’s successes and challenges, the following implications for practice emerge for future comprehensive nutrition education program development and delivery:

- Prioritize effective collaboration with school and community partners, ensuring that each partners’ contributions, goals, limitations, and needs are identified and addressed through the program. Programming that minimizes the need for and role of partners is likely to encounter additional barriers, as well as miss meaningful opportunities to positively impact community health and wellbeing.
- Prioritize the hiring of educators who reflect the youth population and have shared lived experiences, as individuals who live, work, and play in the site communities have a robust and personal understanding of the needs and strengths of the participants, as well as the environments and systems that impact health and wellbeing.
- Engage youth, staff, and community partners in decision-making processes to foster ownership and empowerment within the program. Youth, staff, and community partners should be incorporated into decision-making roles to ensure significant and meaningful contributions to program content development and delivery.
- Recognize and harness the potential of youth as agents of change, empowering them to educate peers, families, and communities on health-related topics. Prior research highlights how youth can positively influence familial and community behaviors [41,44,45]. Training and supporting youth to teach others build their nutrition and physical activity knowledge, while supporting skill development to prepare them for future community-building, civic engagement, and college/career readiness.
- Diversify funding sources beyond SNAP-Ed to enable flexibility and innovation in program design and implementation. Even small amounts of diversified funding enable the program to be responsive to the needs of community members and to develop creative solutions that are not allowable per USDA guidelines.

## Figures and Tables

**Table 1:** Taste Test Tool results by year.

Academic Year	No. of Classes	No. of Students	% of Students Trying This Food for the First Time	% of Students Willing to Eat This Food Again	% of Students Willing to Ask for This Food at Home
2014/15	117	3420	39%	73%	69%
2015/16	48	1357	35%	79%	71%
2016/17	39	1067	36%	69%	63%
2017/18	32	831	59%	76%	71%
2018/19	21	540	56%	69%	65%

**Table 2:** Teacher Observation Tool results by year.

Academic Year	No. of Classes	No. of Students	% of Teachers Reporting That More Students Can Identify Healthy Food Choices at the End of the School Year	% of Teachers Reporting That More Students Are Willing to Try New Foods at School at the End of the School Year	% of Teachers Reporting That More Students Choose Fruits and Vegetables at the End of the School Year
2014/15	79	2378	96%	96%	75%
2015/16	44	1371	100%	95%	78%
2016/17	59	1684	98%	86%	65%
2017/18	45	1264	98%	95%	74%
2018/19	33	810	100%	97%	63%

**Table 3:** EATS Results by Year.

Academic Year	% of Students Who Reported an Increase in Overall Fruit Consumption	% of Students Who Reported an Increase in Overall Vegetable Consumption	% of Students Who Reported a Decrease in Consumption of Sweetened Beverages	% of Students Who Reported an Increase in the Number of Days They Engaged in 60+ min of Physical Activity Last Week
2021/22	36% (*n* = 91)	28% (*n* = 91)	No data	63% (*n* = 91)
2022/23	37% (*n* = 133)	35% (*n* = 121)	44% (*n* = 122)	52% (*n* = 127)

**Table 4:** Teacher Observation Tool results by year.

Academic Year.	N	Compared to the Beginning of the Year, I Now Make My Own Healthier Food Choices	Compared to the Beginning of the Year, I Now Remind Families to Bring Healthy Snacks	Compared to the Beginning of the Year, I Now Encourage the Students to Be Active	Compared to the Beginning of the Year, I Now Offer Students Healthy Choices
2014/15	79	64%	59%	77%	57%
2015/16	41	66%	66%	73%	71%
2016/17	51	66%	98%	61%	59%
2017/18	42	55%	69%	79%	69%
2018/19	33	68%	61%	68%	65%
2022/23	18	82%	70%	89%	70%

**Table 5:** Number of policy, system, and environmental changes adopted by year.

Academic Year	# of PSE Changes Adopted	# of People Reached
2015/16	30	2908
2016/17	40	8791
2017/18	56	7179
2018/19	31	3647
2019/20	103	39,881
2020/21	25	4121
2021/22	37	1804
2022/23	40	2625
Total	362	70,956

## Data Availability

Some of the raw data supporting the conclusions of this article will be made available by the authors on request. For other data, restrictions apply to the availability of these data. Data were obtained from CalFresh Healthy Living Program Evaluation And Reporting System (PEARS) and are available by request at support@pears.io with the permission of CalFresh Healthy Living, UC.
